# Comparative evaluation of free radical scavenging activity and total metabolite profiles among 30 macrofungi species

**DOI:** 10.1186/s40643-025-00841-4

**Published:** 2025-02-21

**Authors:** Tetiana Krupodorova, Victor Barshteyn, Yusufjon Gafforov, Milena Rašeta, Tetiana Zaichenko, Yaroslav Blume

**Affiliations:** 1 Department of Plant Food Products and Biofortification, Institute of Food Biotechnology and Genomics of the National Academy of Sciences of Ukraine, 2a Baidy-Vyshnevetskoho Str., Kyiv, 04123 Ukraine; 2 Central Asian Center for Development Studies, New Uzbekistan University, Tashkent, 100007 Uzbekistan; 3 Department of Chemistry, Biochemistry and Enviromental Protection, Faculty of Science, University of Novi Sad, Novi Sad, 21000 Serbia; 4 Department of Genomics and Molecular Biotechnology, Institute of Food Biotechnology and Genomics of the National Academy of Sciences of Ukraine, 2a Baidy-Vyshnevetskoho Str, Kyiv, 04123 Ukraine

**Keywords:** Mycelium, Culture broth, Media, DPPH, Phenolic compounds, Secondary metabolites, Endopolysaccharides, Exopolysaccharides, Extracts, Submerged cultivation

## Abstract

The high antioxidant content of mushrooms such as polyphenols, polysaccharides, carotenoids, ergothioneine, glutathione, vitamins, and other compounds, has sparked interest in their potential use in preventive and therapeutic medicine. Therefore, this study aimed to evaluate the antioxidant potential of various macrofungi by measuring 1,1-diphenyl-2-picryl-hydrazyl (DPPH) radical scavenging activity and quantifying two classes of compounds: phenolics (TPC) and polysaccharides, both endo- (IPS) and exopolysaccharides (EPS). Species-specific abilities to produce mycelium, polysaccharides, and polyphenols were observed under submerged cultivation without agitation over 14 days. Mycelium productivity (P_M_) ranged significantly from 292 ± 11 to 1110 ± 37 mg·L⁻^1^·day⁻^1^, with the highest values observed in *Ophiocordyceps sinensis* (1110 ± 37 mg·L⁻^1^ ·day⁻^1^), *Pleurotus djamor* (1090 ± 14 mg·L⁻^1^·day⁻^1^), and *Cordyceps militaris* (1080 ± 90 mg· L⁻^1^·day⁻^1^). The DPPH inhibition values of the extracts varied from 4.30 ± 0.20 to 87.9 ± 0.80%, while TPC ranged from 0.35 ± 0.10 to 34.6 ± 0.80 mg GAE/g d.w. *Lentinula edodes* was the best source of produced IPS (10.3 ± 0.35 g·L^−1^), while *Hypsizygus marmoreus* exhibited the highest EPS content (2.24 ± 0.30 g·L^−1^). Overall, IPS levels across species were higher, ranging from 1.56 to 10.3 g·L^−1^. In half of the fungal species, the mycelium exhibited superior antioxidant activity compared to culture broth. However, in the majority (66.7%), TPC was higher in the culture broth than in the fungal mycelium. Distinct scavenging abilities and metabolite profiles were observed among different isolates. Notably, the mycelium of *Fomitopsis pinicola* and *L. edodes* showed the highest DPPH inhibition and TPC. The antioxidant activity and TPC varied depending on the culture medium, and solvents used. The highest DPPH inhibition (90%) and TPC (38.5 ± 0.40 mg GAE/g d.w.) were observed in the methanol (MeOH) extract of *F. pinicola* mycelium grown in Sabouraud dextrose medium. Similarly, MeOH and ethyl acetate (EtOAc) extracts demonstrated strong DPPH inhibition (over 87%), while the maximum TPC (40.0 ± 0.60 mg GAE/g d.w.) was detected in the water (H_2_O) extract of *L. edodes* mycelium cultivated in glucose-peptone-yeast medium. A positive correlation was found between TPC and antioxidant activity, with Pearson’s coefficient of 0.6615 for fungal mycelium, 0.1192 for culture broth, and 0.8924 and 0.7143 for *F. pinicola* and *L. edodes*, respectively. Both fungal mycelium and culture broth exhibit significant potential as natural antioxidants. These can be applied in nutraceuticals, food products, and nutritional supplements, offering diverse health benefits. Additionally, this study is a pioneering exploration of *Auriporia aurea*, *Hohenbuehelia myxotricha*, *Lepista luscina*, *Oxyporus obducens*, and *Pseudospongipellis litschaueri*, highlighting their largely unexplored potential for antioxidant applications.

**Graphical Abstract:**

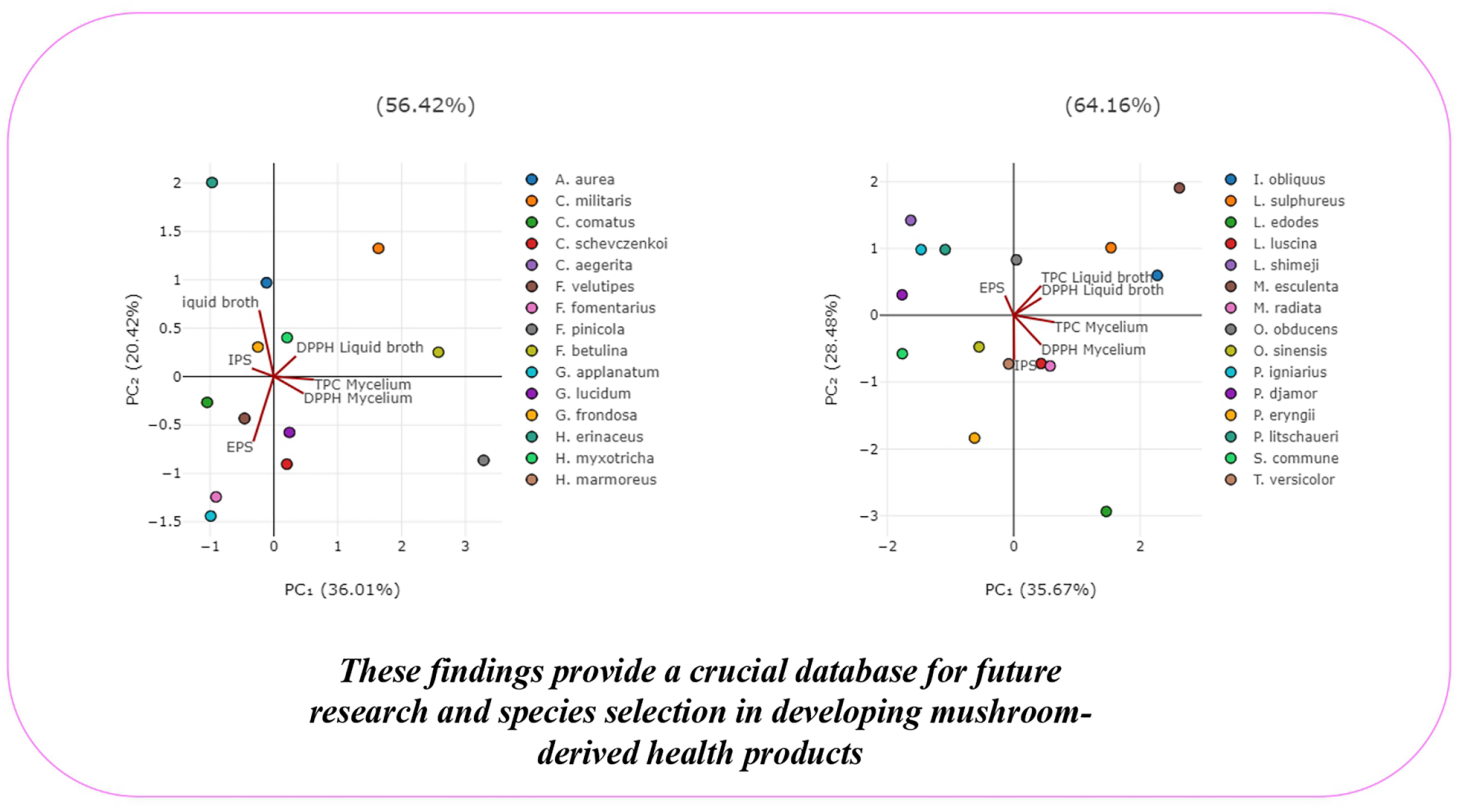

**Supplementary Information:**

The online version contains supplementary material available at 10.1186/s40643-025-00841-4.

## Highlights

1. Mushrooms are rich in antioxidants, aiding disease prevention and health.
2. *Lentinula edodes* excels in producing endopolysaccharides (IPS) at 10.32 g·L^−1^.
3. *Fomitopsis pinicola* shows high DPPH inhibition (90%) in methanol extracts.
4. Mycelium of *F. pinicola* and *L. edodes* shows strong antioxidant potential.
5. Ethyl acetate and methanol extracts reveal valuable antioxidant properties.

**Supplementary Information:**

The online version contains supplementary material available at 10.1186/s40643-025-00841-4.

## Introduction

The increase in reactive oxygen (ROS) and nitrogen (RNOS) species induces oxidative stress, which results in cellular damage and metabolic dysregulation. This process triggers inflammation, apoptosis, DNA damage, lipid peroxidation, and mitochondrial dysfunction, contributing to disorders, including metabolic cardiovascular diseases, Alzheimer’s and Parkinson’s diseases, premature aging, and certain cancers (Kozarski et al. 2015; Sies 2020; Kıran et al. 2023). Antioxidants play a crucial role in mitigating free radical damage by neutralizing ROS and repairing ROS-induced harm, thereby forming a robust antioxidant defense system. Natural antioxidants, which are characterized by low toxicity and minimal side effects, hold significant potential for preventing and managing such diseases (Kozarski et al. 2015; Azieana et al. 2017).

Mushrooms, highly diverse and biochemically potent organisms, have been consumed for centuries for their nutritional and medicinal properties (Tešanović et al. 2017; Suleiman et al. 2022; Gafforov et al. 2023). However, their medicinal and nutraceutical potential remains underutilized in modern markets (Dudekula et al. 2020). Recently, mushrooms have garnered attention as a natural source of antioxidants (Karaman et al. 2022; Suleiman et al. 2022), capable of enhancing antioxidant defenses and reducing oxidative stress through dietary supplementation (Kozarski et al. 2015). Numerous in vitro studies have supported their effectiveness (Rašeta et al. 2016, 2021, 2024a, b; Tešanović et al. 2017; Mišković et al. 2021; Krupodorova et al. 2022; Krsmanović et al. 2023).

Mushrooms, renowned for their polysaccharides, are also abundant in secondary metabolites, including antioxidant compounds such as polyphenols, carotenoids, unsaturated fatty acids, vitamins, enzymes, and cofactors. These compounds effectively neutralize free radicals responsible for oxidative damage, terminating radical chain reactions during lipid oxidation in foods (Karaman et al. 2022). Their high antioxidant content has spurred interest in their application in preventive and clinical medicine (Munteanu and Apetrei 2021).

Additionally, mushrooms present a cost-effective and nutritious means of addressing malnutrition while generating valuable biomass (Dudekula et al. 2020). Submerged fermentation is an efficient method for producing biomass and bioactive metabolites, reducing contamination risk and production time. However, further in vitro and in vivo research is required to fully realize their potential (Dudekula et al. 2020).

Compared to naturally growing fruiting bodies, mycelium produced through submerged fermentation exhibits more consistent antioxidant content, minimizing variability (Wang et al. 2020; Boonthatui et al. 2021; Hu et al. 2023). Increasing interest extends beyond fruiting bodies and mycelia to include culture broths, which also possess significant bioactive properties (Tešanović et al. 2017).

However, antioxidant activity varies widely among fungal species, strains, and forms (fruiting bodies, mycelium, culture broth) (Jeena et al. 2014; Awala and Oyetayo 2015; Nowacka et al. 2015; Angelini et al. 2019; Börühan Çetin et al. 2021; Suleiman et al. 2022). Despite their potential, limited studies have examined the antiradical activity of macrofungal culture broths, highlighting the need for further investigation (Heleno et al. 2012; Soulem et al. 2017; Jiamworanunkul 2019; Regeda et al. 2021; Börühan Çetin et al. 2021; Atamanchuk and Bisko 2023).

Fungal mycelium used in research can be sourced from wild mushrooms collected from different regions (Dong and Yao 2008; Kalyoncu et al. 2010), pharmaceutical companies (Wang et al. 2015a, b; Chang 2022), or institutional collections (Mau et al. 2004; Balan et al. 2010; Chang 2022; Angelini et al. 2023). Submerged cultivation provides a controlled biotechnological method. It is used for obtaining mycelial biomass and producing antioxidants, including polysaccharides and polyphenols, in basidiomycetes (Mišković et al. 2021).

State-supported collections, such as Ukraine’s Mushroom Culture Collection (IBK), provide insights into endangered species and screening fungi for biotechnological applications (Lomberg et al. 2015; Bisko et al. 2018a, b, c; Mykchaylova et al. 2019).

This study aims to provide a comparative overview of the in vitro free radical scavenging potential and total metabolite contents of the mycelium and culture broth of 30 species from the IBK culture collection:

- Quantifying and comparing the total phenolic content (TPC) and total endo- (IPS) and exopolysaccharide (EPS) contents of the 30 species using advanced analytical techniques and assessing mycelium productivity.
- Evaluating and comparing the free radical scavenging capacity of 30 macrofungi species using the DPPH assay.
- Correlating the free radical scavenging activity with specific metabolites or metabolite classes to identify the key antioxidant compounds.
- Identifying the top-performing species with the highest free radical scavenging potency and richest metabolite diversity for further investigation as natural antioxidant sources.

## Materials and methods

### Fungal samples

Investigation of 30 fungal species (*Auriporia aurea* (Peck) Ryvarden 5048, *Coprinus comatus* (O.F. Müll.) Pers. 137, *Cordyceps militaris* (L.) Fr. 1862, *Crinipellis schevczenkoi* Bukhalo 31, *Cyclocybe aegerita* (V. Brig.) Vizzini 1853, *Flammulina velutipes* (Curtis) Singer 1878, *Fomes fomentarius* (L.) Fr. 355, *Fomitopsis betulina* (Bull.) B.K. Cui, M.L. Han &Y.C. Dai 327, *F. pinicola* (Sw.) P. Karst. 1523, *Ganoderma applanatum* (Pers.) Pat. 1701, *G. lucidum* (Curtis) P. Karst. 1900, *Grifola frondosa* (Dicks.) Gray 976, *Hericium erinaceus* (Bull.) Pers. 970, *Hohenbuehelia myxotricha* (Lev.) Singer 1599, *Hypsizygus marmoreus* (Peck) H.E. Bigelow 2006, *Inonotus obliquus* (Fr.) Pilát. 1877, *Laetiporus sulphureus* (Bull.) Murrill 352, *Lentinula edodes* (Berk.) Pegler 502, *Lepista luscina* (Fr.) Singer 64, *Lyophyllum shimeji* (Kawam.) Hongo 1662, *Mensularia radiata* (Sowerby) Lázaro Ibiza 1566, *Morchella esculenta* (L.) Pers. 1953, *Ophiocordyceps sinensis* (Berk.) G.H. Sung, J.M. Sung, Hywel-Jones & Spatafora 1928, *Oxyporus obducens* (Pers.) Donk 5085, *Phellinus igniarius* (L.) Quél. 1578, *Pleurotus djamor* (Rumph. ex Fr.) Boedijn 1526, *P. eryngii* (DC.) Quél. 2015, *Pseudospongipellis litschaueri* (Lohwag) Y.C. Dai & Chai G. Wang 5312, *Schizophyllum commune* Fr. 1768, *Trametes versicolor* (L.) Lloyd 353) were carried out. Pure cultures of 30 selected macrofungal species were kindly obtained from the Mushroom Culture Collection (IBK) at the M. G. Kholodny Institute of Botany of the National Academy of Sciences of Ukraine (Bisko et al. 2021). Fungal cultures were stored at 4 °C on malt extract agar slants.

### Macrofungi cultivation

Pieces of fungal mycelium from agar slants were transferred to Petri dishes containing glucose peptone yeast agar medium (GPYA, pH 6.0) with the following composition per liter: 25.0 g glucose, 3.0 g yeast extract, 2.0 g peptone, 1.0 g K_2_HPO_4_, 1.0 g KH_2_PO_4_, 0.25 g MgSO_4_⋅7H_2_O, and 20.0 g agar. This agar-free medium (GPYB) and Sabouraud dextrose broth (SDB, HiMedia, India, pH 5.6) were autoclaved (Sterilizer VK-75-01, Kyiv, Ukraine) for 15 min at 121 °C. The inoculated liquid media, each inoculated with three mycelial plugs of 8 mm diameter from each culture, were grown under static conditions (without agitation) in the dark in thermostat (TC-80, Kyiv, Ukraine) at 25 °C for 14 days.

The mycelium was separated from the medium by filtration using Whatman No. 4 filter paper, then rinsed with distilled water (dH_2_O) and dried at 85 °C, until a consistent weight was achieved. The fungal growth (biomass) of the selected species *L. edodes* and *F. pinicola* during cultivation was measured as the mycelial biomass (g·L⁻^1^) in terms of absolute dry weight (a.d.w.).

Mycelium productivity (P_M_, mg·L⁻^1^·day⁻^1^) was calculated using the formula (Nayak et al. 2013):

1 $${\text{P}}_{{\text{M}}} = {\text{C}}_{{\text{M}}} \times {1}000/{\text{t}},$$

where C_M_ (g∙L^−1^) is the mycelium concentration at the end of the cultivation. t is the duration of the cultivation, days.

### Preparation of the extracts

The mycelium from each of the macrofungal species was separated from the culture broth by filtration (Whatman No. 4 filter paper, Whatman plc, UK) and then washed three times with dH_2_O. The washed mycelium was dried at 60 °C (Snol-58/350, UMEGA, Lithuania) and ground into a fine powder using an electric grinder (VHC-150, Kyiv, Ukraine).

For extraction, 1 g of fungal mycelium was mixed with 10 mL of ethyl acetate (EtOAc) and incubated at room temperature on an orbital shaker (Santarius, Göttingen, Germany) at 100 rpm for 72 h. Additionally, some species (*Lentinula edodes* and *Fomitopsis pinicola*) were also extracted using methanol (MeOH), ethanol (EtOH), dH_2_O, and water–ethanol solutions (50% EtOH and 70%EtOH) following the same procedure. After extraction, the mycelial extracts were centrifuged at 9000 rpm at 4 °C for 10 min (Eppendorf MiniSpin, Hamburg, Germany), and the supernatants were collected for further analyses.

The culture broth was subjected to liquid–liquid extraction with EtOAc in a 1:2 ratio at room temperature, with stirring at 100 rpm for 72 h. The fractions were separated using a dividing funnel (VD-1-1000, Labexpert, Kyiv, Ukraine), the EtOAc fraction was evaporated under reduced pressure using a vacuum rotary evaporator (RVO-64, Czechoslovakia) at 40 °C. The resulting residues were dissolved in a solvent (1:1 w/v) and prepared for further analyses.

### Quantification of the total phenolic content (TPC)

The TPC was determined using the Folin–Ciocalteu method described by Reis et al. (2012), a highly sensitive technique with a broad detection range, suitable for quantifying diverse phenolic compounds in complex biological samples. Briefly, 1 mL of the extract sample was mixed with 0.5 mL Folin–Ciocalteu’s reagent (diluted 1:10 with dH_2_O), followed by the addition of 4 mL with sodium carbonate (75 g·L^−1^). The mixture was vortexed for 15 s (Fisher Vortex Genie 2, New York, USA) and incubated at 40 °C for 30 min. The absorbance was measured at 765 nm using a UV–Vis spectrophotometer (UV-1800 PC, Shanghai, China). A control solution containing all reagents except the fungal extract. TPC was quantified using a gallic acid (GA) calibration curve, and results were expressed as milligrams of GA equivalents per gram of dry weight sample (mg GAE/g d.w.).

The TPC productivity P_TPC_ (mg·L^−1^·day^−1^) was calculated by using the following equation:

2 $${\text{P}}_{{{\text{TPC}}}} = {\text{ C}}_{{{\text{TPC}}}} /{\text{t}},$$

where C_TPC_ (mg∙L^−1^) is the TPC in the mycelium at the end of the cultivation. t is the duration of the cultivation, days.

### Quantification of the endopolysaccharides (IPS)

The separated mycelium was washed with dH_2_O and dried at 60 °C. It was then crushed and suspended in dH_2_O at a 1:10 ratio. The suspension was boiled in a water bath (UOSlab BH 09, Kiyv, Ukraine) for 18 h. The cytoplasmic content of the mycelium in dH_2_O was removed by centrifugation at 5.000×*g* for 10 min. The resulting mixture was concentrated using a rotary evaporator at 60 °C. The concentrated solution was re-deposited by adding cold EtOH (96%, 1 volume), stirred, and left overnight at 4 °C. The resulting precipitate, representing IPS, was collected by centrifugation and dialyzed against dH_2_O for 3 days. The dialyzed IPS was further precipitated with EtOH (96%, 2 volumes), washed with ether and acetone, and finally dried at 40 °C. The chosen IPS quantification method ensures high specificity, efficiently isolating intracellular polysaccharides with minimal contamination. Sequential steps of washing, boiling, and precipitation effectively removed unwanted cytoplasmic components, while dialysis and EtOH precipitation maximized purity. The total yield of IPS was determined using a gravimetric method, which provides a straightforward and reliable means of quantification. The IPS yield was expressed as a percentage of the absolute dry biomass (Mykchaylova et al. 2023).

The IPS productivity P_IPS_ (mg·L^−1^·day^−1^) was calculated by using the following equation:

3 $${\text{P}}_{{{\text{IPS}}}} = {\text{C}}_{{{\text{IPS}}}} /{\text{t}},$$

where C_IPS_ (mg·L^−1^) is the IPS concentration in the mycelium at the end of the cultivation. t is the duration of the cultivation, days.

### Quantification of the exopolysaccharides (EPS)

After 14 days of cultivation, the mycelium biomass of macrofungi was filtered through Whatman filter paper No. 2 (Whatman International Ltd., Maidstone, UK), and the culture broth was evaluated for EPS production. EPS were obtained by precipitating the culture broth with cold EtOH (96%, 4 volumes). The mixture was vigorously stirred and left overnight at 4 °C. The resulting precipitate was separated by centrifugation at 7.245×*g* for 10 min, dialyzed against dH_2_O, and dried at 60 °C until a constant weight was achieved. This method for quantifying EPS was chosen for its reliability and accuracy in isolating and measuring polysaccharides from culture broth. Ethanol precipitation is an efficient and widely used technique for concentrating polysaccharides, while dialysis effectively removes small impurities. The crude EPS in the precipitate was quantified using the phenol–sulfuric acid method (Dubois et al. 1956), a well-established and sensitive assay for carbohydrate quantification that provides precise estimation of EPS yield using glucose as a standard. The crude EPS yield was calculated and expressed as grams of EPS per liter of culture medium.

The EPS productivity P_EPS_ (mg·L^−1^·day^−1^) was calculated by using the following equation:

4 $${\text{P}}_{{{\text{EPS}}}} = {\text{C}}_{{{\text{EPS}}}} \times {1}000/{\text{t}},$$

where C_EPS_ (g·L^−1^) is the EPS concentration in the liquid broth at the end of the cultivation. t is the duration of the cultivation, days.

### Free-radical scavenging capacity

The free-radical scavenging capacity of the prepared fungal extracts was evaluated using the 1,1-diphenyl-2-picryl-hydrazyl (DPPH) assay, a widely used method known for its simplicity and the clear visualization of the color change from purple to yellow. An aliquot of each fungal extract (100 μL) was mixed with 2900 μL of DPPH solution (120 μM in MeOH) and incubated in the dark at 37 °C for 30 min. The absorbance was measured at 517 nm using MeOH as a blank (Espín et al. 2000). A control sample with only MeOH and DPPH (without fungal extract) was used to calculate the radical scavenging activity. The inhibition of DPPH free radicals (%) was calculated according the following equation:

5 $${\text{Inhibition }}\left( \% \right) = \left[ {\left( {{\text{A}}_{{{\text{control}}}} - {\text{A}}_{{{\text{sample}}}} } \right)/{\text{A}}_{{{\text{control}}}} } \right] \times {1}00,$$

where A_control_ is the absorbance of the control (mixture of MeOH and DPPH reagent without fungal extract). A_sample_ is the absorbance of the sample (mixture of fungal extract and DPPH reagent).

### Statistical analysis

All assays were performed in triplicate, and the results are presented as the mean ± standard deviation (SD) of three replicates. Statistical analysis was conducted using one-way ANOVA (Statistica 11.5, StatSoft Inc., USA), followed by Fisher's LSD test. Differences were considered statistically significant at p < 0.05. Correlations between radical scavenging activity and TPC, IPS and EPS were evaluated using Pearson correlation coefficients, categorized according to Evans` categorization (1996). Principal Component Analysis (PCA) was performed using the online statistical software available at Statistics Kingdom. (2024).

## Results and discussion

The search for new natural antioxidants remains a significant area of modern research. Many fungi demonstrate the ability to neutralize free radicals, which contributes to their therapeutic effects (Sánchez 2017). In this study, we investigated the antioxidant potential of a diverse set of edible and medicinal macrofungi, including well-studied species such as *G.  lucidum*, *L. edodes*, and *P.  eryngii*, known for their phenolic and polysaccharide content, as well as underexplored species like *A. aurea, O.  obducens,* and *P.  litschaueri*.

Macrofungi were selected based on their taxonomic and ecological diversity to ensure a wide range of biochemical and metabolic profiles. Well-studied species, recognized for their therapeutic and nutritional properties, provide insights into the effects of strain-specific metabolites and cultivation conditions (Kalyoncu et al. 2010; Mišković et al. 2021). On the other hand, poorly studied fungi, such as *Crinipellis schevczenkoi* and *Hohenbuehelia myxotricha*, offering potential for discovering novel bioactive compounds, as suggested by prior findings on their anticancer, antibacterial, and antagonistic activities (Krupodorova et al. 2016, 2023, 2024).

By including both well-studied and underexplored species, this research highlights the importance of cultivation conditions including nutrient medium composition and extraction solvents in determining the antioxidant properties of fungal mycelium and culture liquids (Cui et al. 2005; Jang et al. 2016; Tešanović et al. 2017). Such an approach ensures a comprehensive understanding of their antioxidant capacities and potential applications in therapeutic and nutraceutical contexts.

### Productivity of mycelium and total metabolites

The use of mycelial cultures of macrofungi for experimental and medical applications is a promising and reliable method, enabling efficient production of fungal biomass and its metabolites (Sułkowska‐Ziaja et al. 2018). Variations in the content of bioactive compounds in fruiting bodies collected from natural environments may result from genetic differences between populations, as well as differences in habitats and environmental conditions during growth and development. However, under the controlled, reproducible conditions of mycelial cultures, the differences in the amounts of bioactive compounds, such as polysaccharides, terpenoids, steroids, and phenolic compounds, are significantly reduced (Sulkowska‐Ziaja et al. 2018).

The growth of mycelia for each tested species was conducted in flasks with GPYB medium over a 2-week period (optimal cultivation period for most studied species) using submerged cultivation without agitation. All tested species were able to grow in the studied medium, although mycelial growth and metabolite production varied significantly depending on the fungal species. The results are presented in Table 1 as mean ± SD. During this 14-day period, the highest mycelial productivity (P_M_) was observed in *O. sinensis* (1110 ± 37 mg·L^−1^·day^−1^), *P. djamor* (1090 ± 14 mg·L^−1^·day^−1^), and *C. militaris* (1080 ± 90 mg·L^−1^·day^−1^). The lowest growth was noticed in *L. sulphureus* (228 ± 18 mg·L^−1^·day^−1^), *H. erinaceus* (242 ± 19 mg·L^−1^·day^−1^), *I. obliquus* (285 ± 16 mg·L^−1^·day^−1^), and *L. luscina* (292 ± 11 mg·L^−1^·day^−1^). Compared to our results, Ghatnur et al. (2015) reported lower P_M_ for *O. sinensis* (377.86 mg·L^−1^·day^−1^) after 14 days of cultivation. Similarly, Borges et al. (2014) also noted lower P_M_ for *P. djamor* mycelium (571.42 mg·L^−1^·day^−1^) during the same cultivation period. Wang et al. (2019) recorded similar P_M_ for *C. militaris* (1000 mg·L^−1^·day^−1^) after 14 days of submerged cultivation under optimized conditions. Similarly, Umeo et al. (2015) observed a high level of P_M_ (1190 ± 52 mg·L^−1^·day^−1^) for *Lentinus crinitus*. Additionally, the P_M_ of *L. edodes* (369 ± 45 mg·L^−1^·day^−1^) and *S. commune* (868 ± 24 mg·L^−1^·day^−1^) observed in their study is comparable to our results for these species (Table 1).

**Table 1:** Productivity of mycelium and total metabolites of macrofungi on the14th day of cultivation

Macrofungi species	P_M_ (mg·L^−1^·day^−1^)		P_TPC_ (mg·L^−1^·day^−1^)		P_IPS_ (mg·L^−1^·day^−1^)		P_EPS_ (mg·L^−1^·day^−1^)	
*A. aurea*		842 ± 70		0.72 ± 0.03		41.7 ± 0.3		40.0 ± 0.2
*C. militaris*		1080 ± 90		0.59 ± 0.02		58.1 ± 0.5		37.1 ± 0.1
*C. comatus*		671 ± 28		0.15 ± 0.01		43.9 ± 0.2		55.7 ± 0.3
*C. schevczenkoi*		507 ± 15		0.59 ± 0.04		33.2 ± 0.2		94.3 ± 0.4
*C. aegerita*		621 ± 38		0.02 ± 0.01		60.1 ± 0.6		12.9 ± 0.1
*F. velutipes*		635 ± 35		0.51 ± 0.02		34.3 ± 0.1		132.9 ± 0. 6
*F. fomentarius*		792 ± 66		0.08 ± 0.03		30.0 ± 0.4		147.1 ± 0.7
*F. betulina*		578 ± 86		1.1 ± 0.5		14.1 ± 0.1		12.9 ± 0.1
*F. pinicola*		635 ± 89		1.4 ± 0.6		28.2 ± 0.2		22.1 ± 0.2
*G. applanatum*		850 ± 34		0.08 ± 0.04		30.7 ± 0.4		138.6 ± 0.4
*G. lucidum*		814 ± 50		0.28 ± 0.03		38.4 ± 0.1		54.3 ± 0.2
*G. frondosa*		435 ± 2		0.23 ± 0.06		30.5 ± 0.3		30.7 ± 0.1
*H. erinaceus*		242 ± 19		0.12 ± 0.01		8.5 ± 0.1		20.0 ± 0.1
*H. myxotricha*		442 ± 48		0.36 ± 0.31		33.4 ± 0.5		31.4 ± 0.1
*H. marmoreus*		321 ± 9		0.08 ± 0.02		28.3 ± 0.1		160 ± 0.3
*I. obliquus*		285 ± 16		1.37 ± 0.10		9.6 ± 0.1		57.1 ± 0.1
*L. sulphureus*		228 ± 18		1.1 ± 0.5		11.0 ± 0.1		58.6 ± 0.1
*L. edodes*		428 ± 13		1.4 ± 0.3		45.1 ± 0.5		50.0 ± 0.1
*L. luscina*		292 ± 11		0.72 ± 0.04		13.4 ± 0.2		22.9 ± 0.1
*L. shimeji*		885 ± 26		0.05 ± 0.02		13.8 ± 0.2		65.7 ± 0.1
*M. esculenta*		642 ± 25		1.8 ± 0.7		19.6 ± 0.1		48.6 ± 0.1
*M. radiata*		492 ± 98		1.1 ± 0.6		20.7 ± 0.2		63.6 ± 0.1
*O. obducens*		500 ± 22		0.40 ± 0.06		22.9 ± 0.1		34.3 ± 0.1
*O. sinensis*		1110 ± 37		0.86 ± 0.03		70.9 ± 0.8		90.0 ± 0.6
*P. igniarius*		707 ± 19		0.11 ± 0.04		26.9 ± 0.2		77.1 ± 0.4
*P. djamor*		1090 ± 14		0.05 ± 0.02		49.5 ± 0.3		60.0 ± 0.2
*P. eryngii*		785 ± 87		0.57 ± 0.03		65.9 ± 0.6		8.6 ± 0.1
*P. litschaueri*		621 ± 23		0.11 ± 0.05		31.1 ± 0.4		90.0 ± 0.3
*S. commune*		785 ± 18		0.16 ± 0.01		52.2 ± 0.6		52.9 ± 0.1
*T. versicolor*		564 ± 37		0.45 ± 0.01		40.1 ± 0.3		31.4 ± 0.1

Significant differences were determined by one-way ANOVA using the Fisher's LSD test. Data represent the mean ± standard deviation (SD), and differences were considered statistically significant at *p* < 0.05. The following abbreviations are used for the examined parameters: day, day of mycelia growth; P_M_, mycelium productivity; P_TPC_, productivity of total phenolic content; P_IPS_, productivity of endopolysaccharides; P_EPS_, productivity of exopolysaccharides

Of particular interest in fungal cultivation are secondary metabolites, such as polyphenolic compounds (Abdelshafy et al. 2021; Krsmanović et al. 2023). The global market for antioxidants derived from phenolic compounds was valued at USD 1785.5 million in 2022 and is projected to reach USD 2307.6 million by 2029, with a compound annual growth rate (CAGR) of 3.7% LinkedIn. (2024). All tested macrofungi were able to produce phenolic compounds, with significant variation between species (Table 1). The highest P_TPC_ was observed in *M. esculenta* (1.8 ± 0.7 mg·L^−1^·day^−1^), *L. edodes* (1.4 ± 0.3 mg·L^−1^·day^−1^), and *F. pinicola* (1.4 ± 0.6 mg·L^−1^·day^−1^). The lowest P_TPC_ was observed in *C. aegerita* (0.02 ± 0.01 mg·L^−1^·day^−1^), *L. shimeji* (0.05 ± 0.02 mg·L^−1^·day^−1^) and *P. djamor* (0.05 ± 0.02 mg·L^−1^·day^−1^). Reis et al. (2012) reported a lower P_TPC_ (0.26 mg·L^−1^·day^−1^) in *L. edodes* mycelium after 48 days of cultivation. Some phenolic compound profiles were identified in *L. edodes* mycelium (Kała et al. 2021; Wu et al. 2023). To the best of our knowledge, there are no data for the P_TPC_ of *M. esculenta*, although Mau et al. (2004) reported TPC of 3.63 ± 0.31 mg·L^−1^ in *M. esculenta* mycelium.

Polysaccharides, among various valuable primary metabolites, have attracted considerable interest in macrofungi cultivation due to their unique properties and broad application potential. The global market for polysaccharides and oligosaccharides is projected to exceed USD 22 billion, growing at a CAGR of over 5% from 2020 to 2030. All analyzed macrofungi were capable of producing polysaccharides, with significant differences observed depending on the fungal species (Table 1).

The highest productivity of identified polysaccharides was noted for IPS (P_IPS_) in *P. eryngii* (65.9 ± 0.6 mg·L^−1^·day^−1^), *C. aegerita* (60.1 ± 0.6 mg·L^−1^·day^−1^), and *C. militaris* (58.1 ± 0.5 mg·L^−1^·day^−1^). The lowest P_IPS_ values were observed in *H. erinaceus* (8.5 ± 0.1 mg·L^−1^·day^−1^) *I. obliquus* (9.6 ± 0.1 mg·L^−1^·day^−1^), *and L. sulphureus* (11.00 ± 0.1 mg·L^−1^·day^−1^). Diamantopoulou et al. (2014) reported varying P_IPS_ values for *Cyclocybe aegerita* (formerly *Agrocybe aegerita*) under different conditions: 40.0, 241.5, 229.0 mg·L^−1^·day^−1^ on 8, 20, 24 days of cultivation, respectively, under static conditions, and 9.05, 241.67 mg·L^−1^·day^−1^ on 16 and 24 days of cultivation, respectively, under agitated condition. Hsieh et al. (2005) observed higher P_IPS_ values for *C. militaris*, reaching 435.71 and 1.070 mg·L^−1^·day^−1^ on days 7 and 3, respectively, under optimized cultivation medium in a shake flask and fermenter. While there is considerable literature on the isolation and biological properties of *P. eryngii* IPS (Ma et al. 2017, 2020, 2022, 2024; Gong et al. 2022; Vlassopoulou et al. 2022), data on P_IPS_ during submerged cultivation is, to the best of our knowledge, not available.

P_EPS_ production was observed in *H. marmoreus* (160 ± 0.3 mg·L^−1^·day^−1^), with significant amounts also found in *G. applanatum* (138.6 ± 0.4 mg·L^−1^·day^−1^) and *F. velutipes* (132.9 ± 0.6 mg·L^−1^·day^−1^). The lowest P_EPS_ value was found for *P. eryngii* (8.6 ± 0.1 mg·L^−1^·day^−1^), followed by *C. aegerita* and *F. betulina*, both at value of 86 mg·L^−1^·day^−1^. Some of our results for P_EPS_ in these three species were lower compared to other studies conducted under optimized submerged cultivation with agitation. For instance, Chen et al. (2017) reported a higher P_EPS_ (0.024 g·L^−1^·h^−1^) for *H. marmoreus* using a two-stage pH strategy in a glucose-based medium. In our previous study, another strain of *G. applanatum* (1527 IBK) produced 827.27 mg·L^−1^·day^−1^ (Krupodorova 2011). However, the P_EPS_ of our *G. applanatum* strain (1700 IBK) was higher than that of the *G. applanatum* UCC002 strain, which produced 13.57 mg·L^−1^·day^−1^ under submerged agitation cultivation and 16.43 mg·L^−1^·day^−1^ under static condition (Montoya et al. 2013), Additionally, compared to *G. applanatum* KFRI 1646, with P_EPS_ value as 100 mg·L^−1^·day^−1^ under submerged agitation (Lee et al. 2007), our strain showed higher P_EPS_. Furthermore, compared to our *F. velutipes* strain, Wu et al. (2021) observed lower P_EPS_ values in monoculture (40 mg·L^−1^·day^−1^) and in co-culture with *G. lucidum* (15 mg·L^−1^·day^−1^) with 14 days of growth. Moreover, Lung and Huang (2012) reported higher P_EPS_ (58.57 mg·L^−1^·day^−1^) and P_IPS_ (10.99 mg·L^−1^·day^−1^) values for *L. sulphureus* compared to our results.

Notably, species within the same genus, such as *G. applanatum*, *G. lucidum*, *P. eryngii*, and *P. djamor*, exhibited significant differences in TPC and EPS productivity compared to IPS and mycelium growth (Table 1). In contrast, for *F. betula* and *F. pinicola*, only a two-fold difference was observed in P_IPS_ and P_EPS_ values, while P_M_ and P_TPC_ values were not statistically different.

Pearson’s correlation analysis (Table S1) revealed a significant positive correlation between P_M_ and P_IPS_ (r^2^ = 0.6505), a small positive correlation between P_M_ and P_EPS_ (r^2^ = 0.1339), and a small negative correlation between P_M_ and P_TPC_ (r^2^ = -0.2424).

### Mycochemical characterization

#### Total phenolic content (TPC)

**Fig. 1:**
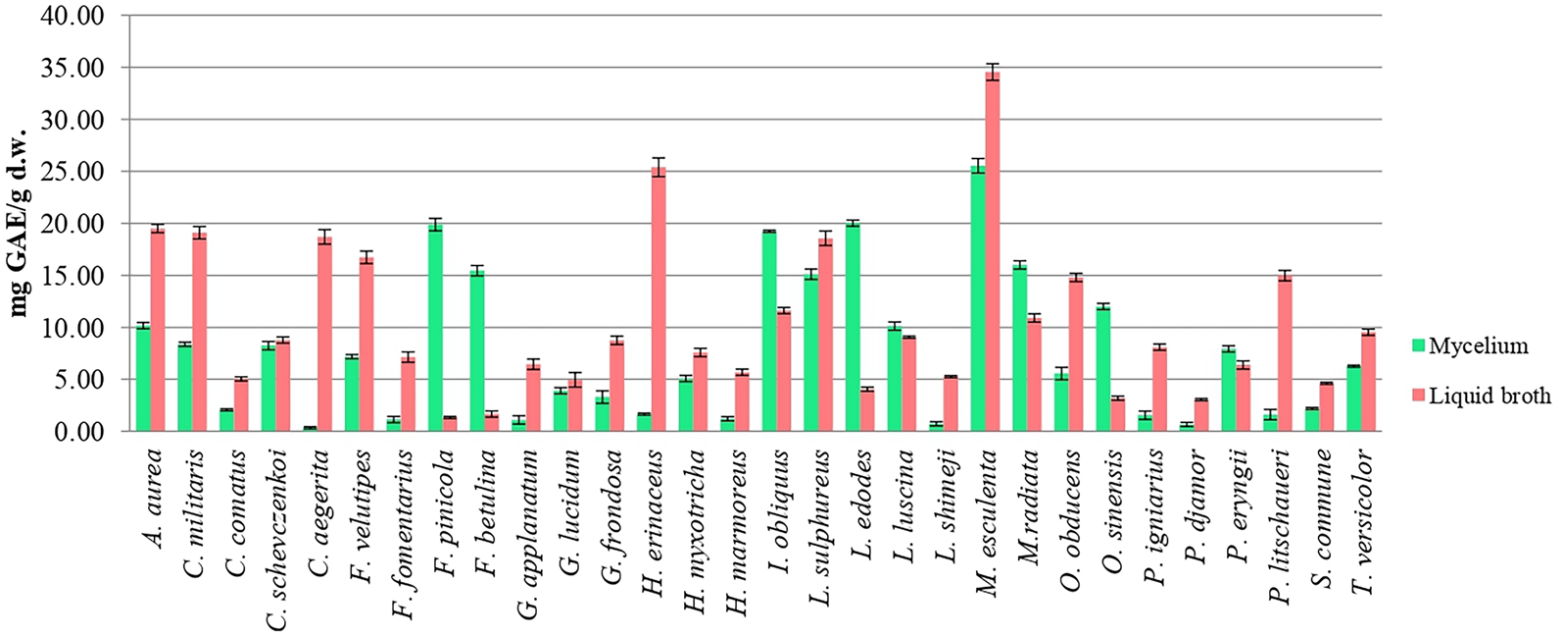
Total phenolic content of ethyl acetate extracts from 30 fungal species. Significant differences were determined by one-way ANOVA using the Fisher's LSD test. Data represent the mean ± standard deviation (SD), and differences were considered statistically significant at *p* < 0.05. The following abbreviations are used for the examined parameters: d.w., dry weight; GAE, gallic acid equivalents

Quantification of TPC in the mycelium of various fungal species provides valuable insights into their antioxidant activities and secondary metabolite production. The TPC values ranged from 0.35 ± 0.10 to 34.6 ± 0.80 mg GAE/g d.w., varying by species and sample type (mycelium or culture broth) (Fig. 1). Our results indicated that for most of the studied fungal species (66.7%), the TPC was higher in the culture broth compared to the fungal mycelium. The highest TPC was observed in *M. esculenta*, with 34.6 ± 0.80 mg GAE/g d.w. in the culture broth and 25.5 ± 0.70 mg GAE/g d.w. in the mycelium, followed by *F. pinicola* (19.9 ± 0.60 mg GAE/g d.w.) and *L. edodes* (20.0 ± 0.30 mg GAE/g d.w.) mycelium, and *H. erinaceus* (25.4 ± 0.99 mg GAE/g d.w.) culture broth. Generally, no statistical difference in TPC was found between culture broth and mycelium in *C. schevczenkoi* and *G. lucidum* (Fig. 1). Additionaly, Table 1 and Fig. 1 confirms that *M. esculenta*, *L. edodes*, and *F. pinicola* exhibit the highest P_TPC_ values, reflecting their abundant secondary metabolites. Significant differences in TPC were observed within genera, such as *F. betulina*, *F. pinicola*, *P. eryngii*, and *P. djamor*. However, among *Ganoderma* species, significant differences in TPC were only noted in the mycelium of *G. applanatum* and *G. lucidum* (Fig. 1).

Screening studies are essential for identifying promising producers of various metabolites and expanding our knowledge of lesser-known species. To the best of our knowledge, there is no information on TPC production in submerged cultivation for *C. aegerita*, *H. myxotricha*, *L. luscina*, *L. shimeji*, *M. radiata*, *O. obducens*, and *P. litschaueri*. In comparison with literature data, Valu et al. (2020) reported a TPC of 23.2 mg GAE/g in *H. erinaceus* mycelium, which is lower than our result but comparable to that in the culture liquid. This finding is consistent with Narmuratova et al. (2023), who reported TPC values for six *H. erinaceus* strains ranging from 2.34 ± 0.05 to 3.15 ± 0.05 mg GAE/g. Similarly, *S. commune* cultured on wheat exhibited the highest phenolic compound production after 8 days (8.56 ± 1.09 mg GAE/g d.w.) (Boonthatui et al. 2021), which was two to four times higher than our result for mycelium or liquid broth samples (Fig. 1). Furthermore, Mishra et al. (2013) reported TPC values for different oyster mushroom species ranging from 3.94 to 21.67 mg tannic acid equivalent (TAE)/g. Among these, *P. eryngii* had the highest TPC (21.67 mg TAE/g of mycelium), followed by *P. djamor* (18.88 mg TAE/g of mycelium), both significantly higher than our findings. Reis et al. (2012) found TPC values of 9.11 ± 0.23 mg GAE/g extract for *P. eryngii* and 12.53 ± 0.30 mg GAE/g extract for *L. edodes* mycelia, which are significantly lower than our results (Fig. 1). Onar et al. (2016) tested 70% EtOH and pure EtOH extracts of *F. pinicola*, reporting TPC values of 278.0 ± 2.9 mg GAE/g extract and 313.2 ± 5.8 mg GAE/g extract, respectively, which are substantially higher than our findings.

Huang et al. (2021) investigated submerged cultivated mycelial biomass over 7 to 14 days, depending on the species, using hot H_2_O extracts from combinations and ratios of sixteen medicinal mushroom species, including *Auricularia auricula-judae*, *C.  militaris*, *C.  comatus*, *F.  velutipes*, *G.  lucidum*, *G.  frondosa*, *H.  erinaceus*, *I.  obliquus*, *L.  edodes*, *P.  eryngii*, and *T.  versicolor*. TPC values in all samples ranged from 15.53 ± 0.23 to 18.88 ± 0.34 mg GAE/g d.w., with the highest observed in a combination of five species (*P. ostreatus*, *G. lucidum*, *A. auricula-judae*, *T. versicolor*, and *L. edodes*), comparable to results from this study. The mycelium of *C. comatus* in this study showed a significantly lower TPC (Fig. 1) compared to the findings of Tešanović et al. (2017) (81.95 ± 2.62 mg GAE/g d.w.). Similarly, the liquid broth from this study exhibited significantly lower amounts of TPC than reported by Tešanović et al. (2017) (69.48 ± 3.51 mg GAE/g d.w.). Mišković et al. (2021) obderved higher TPC in mycelial samples compared to this study, with Serbian and Italian strains of *S. commune* yielding 76.65 ± 1.30 mg GAE/g d.w. and 82.62 ± 0.99 mg GAE/g d.w., respectively, after 14 days of cultivation. Huang et al. (2021) reported TPC values randing from 15.53 ± 0.23 to 18.88 ± 0.34 mg GAE/g d.w. for submerged cultivated mycelial biomass of *C. comatus, C. militaris, F. velutipes, G. lucidum, G. frondosa, H. erinaceus, I. obliquus, L. edodes, P. eryngii*, and *T. versicolor*. Among their mixtures, Spl 5 (*P. ostreatus* (40%), *G. lucidum* (15%), *A. auricula-judae* (15%), *T. versicolor* (15%), and *L. edodes* (15%) achived the highest TPC (18.88 ± 0.34 mg GAE/g), while Spl 6 (*P. ostreatus* (30%), *G. lucidum* (20%), *A. auricula-judae* (20%), *T. versicolor* (15%), and *L. edodes* (15%), had the lowest. These findings suggest that species tested in combinations, as reported by Huang et al. (2021), are more effective in achieving higher TPC compared to individually tested species.

Deshmukh and Lakshmi (2023) reported that H_2_O extracts of *C. militaris* mycelium exhibited a higher TPC (8.50 mg GAE/g), consistent with the results presented in Fig. 1.

Cultivation conditions can effectively enhance TPC in macromycetes. Krsmanović et al. (2023) observed significantly higher TPC in *F. velutipes* after 4 weeks of UV-treated cultivation (58.34 ± 1.70 mg GAE/g d.w.) compared to this study’s 2-week cultivation, while *T. versicolor* results were comparable (15.56 ± 0.30 mg GAE/g d.w.). Their study also reported lower TPC in *F. velutipes* filtrates (14.86 ± 0.55 mg GAE/g d.w.) but fivefold higher TPC in *T. versicolor* filtrates (47.22 ± 0.49 mg GAE/g d.w.) compared to our results. Hung et al. (2020) noted TPC variation in *C. militaris* (1.75 ± 0.07 to 3.74 ± 0.18 mg·mL^−1^) based on cultivation medium, fermentation type, and strain. The addition of *Radix Puerariae* to the cultivated medium significantly increased *S. commune* TPC production to 3731.56 mg GAE/g, compared to 288.35 mg GAE/g in the control medium (Deng et al. 2021). Our previous study (Krupodorova et al. 2024) demonstrated that optimizing culture conditions by adjusting three factors such as temperature, carbon source, and pH resulted in a 2.25- and 2.23-fold increase in TPC production by *F. pinicola* under shaking and static culture conditions, respectively.

This study is the first to report TPC in the mycelium of *L. lucina*, *L. shimeji*, and *M. radiata*, despite existing studies on their fruiting bodies.

These comparisons highlight the variability in TPC across fungal species and emphasize the importance of standardized cultivation and extraction protocols. The observed differences in TPC values among studies may also reflect the genetic diversity of environmental adaptations of fungi.

#### Total polysaccharide contents

As Sivanesan et al. (2022) stated, mushrooms have a long history, dating back to Ancient Egypt, and are widely explored in research and cuisine. Their polysaccharides are valued as nutraceuticals, playing a crucial role in treating various human diseases and disorders. Polysaccharides from the mycelium and liquid broth of macrofungi have demonstrated diverse biological activities, including antioxidant, anti-inflammatory, antitumor, antiviral, anti-hyperlipidemic, anti-hyperglycemic, hepatoprotective, and immunomodulating effects (Zhang et al. 2007; Xiang et al. 2022; Flores et al. 2024). Due to the significant strain-specific characteristics of polysaccharide synthesis in macromycetes (Umeo et al. 2015; Kizitska et al. 2024), identifying promising producers remains a key focus of biotechnological research.

**Fig. 2:**
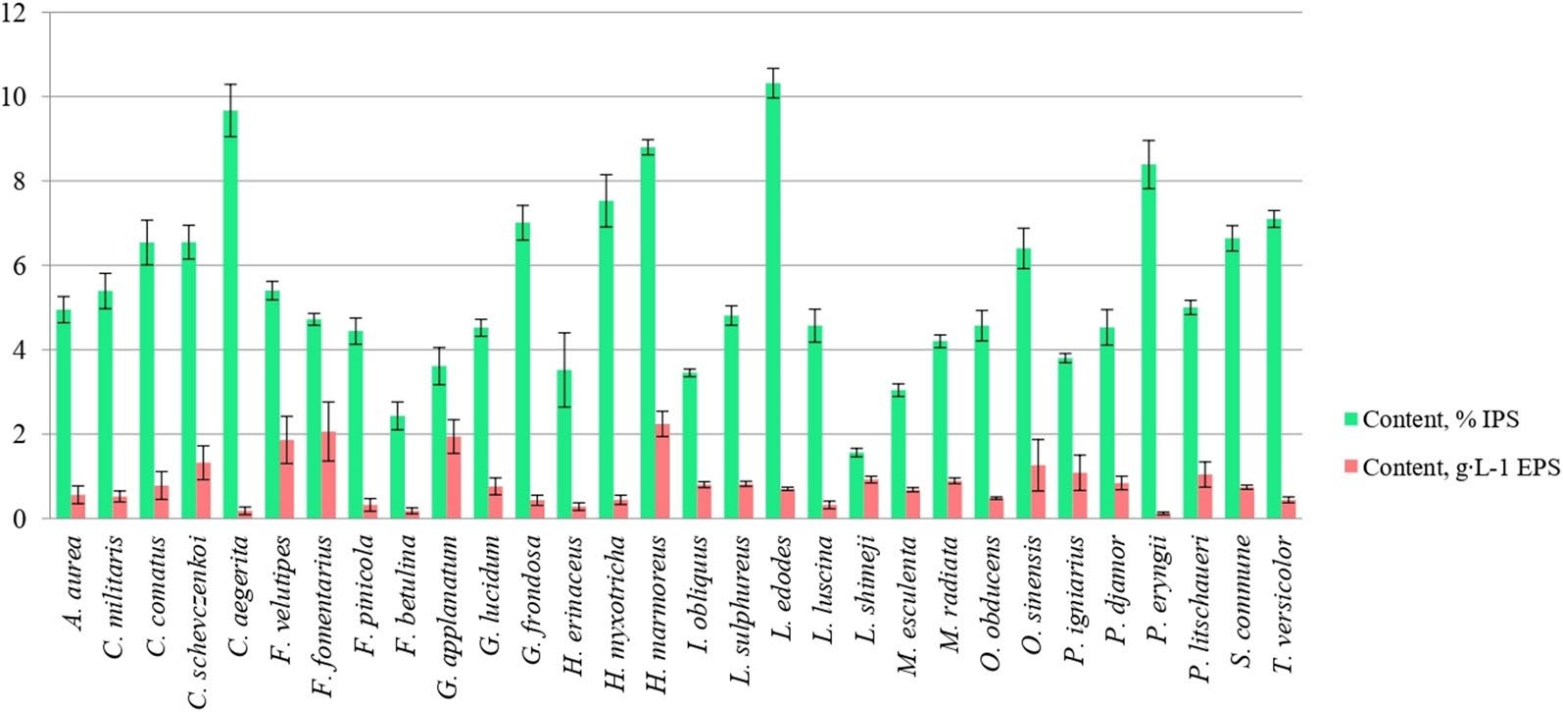
Total contents of endopolysaccharides (IPS) and exoploysacchaides (EPS) in ethyl acetate extracts from 30 fungal species. Significant differences were determined by one-way ANOVA using the Fisher's LSD test. Data represent the mean ± standard deviation (SD), and differences were considered statistically significant at *p* < 0.05. The following abbreviations are used for the examined parameters: IPS, endopolysaccharides; EPS, exoploysacchaides. Values are presented as means ± standard deviation of three replicates (p < 0.05)

In this study, IPS and EPS were quantified in all fungal samples. IPS values in the mycelium ranged from 1.56 ± 0.10% to 10.3 ± 0.35%, with *L. edodes* containing showing the highest amount (10.3 ± 0.35%), followed by *C. aegerita* (9.67 ± 0.62%) and *H. marmoreus* (8.80 ± 0.18%) (Fig. 2). EPS values in the culture broth ranged from 0.12 ± 0.03 g∙L^−1^ to 2.24 ± 0.30 g∙L^−1^, with *H. marmoreus* producing the highest EPS (2.24 ± 0.30 g∙L^−1^), followed by *F. fomentarius* (2.06 ± 0.70 g∙L^−1^) and *G. applanatum* (1.94 ± 0.40 g∙L^−1^) (Fig. 2).

Moreover, Table 1 highlights *C. aegerita* as a significant producer of both IPS and EPS, while *H. marmoreus*, *G. applanatum*, and *F. velutipes* showed the highest P_EPS_ values.

The high IPS and EPS production by edible species like *L. edodes*, *C. aegerita*, and *H. marmoreus* underscores their potential for food and nutraceutical applications (Giavasis 2014). For instance, lentinan from *L. edodes* mycelium has demonstrated antiviral (Ren et al. 2018) and immunomodulating (Turło et al. 2010) activities.

Optimization of the conditions for the submerged cultivation of *L. edodes* has yielded different IPS values: 7.9% (Bisko et al. 2020), 46.91 mg∙g^−1^ (Ameri Shah Reza et al. 2018). Wang et al. (2009) achieved 1.46 g∙L^−1^ IPS, while Chen et al. (2017) observed 1.81 g∙L^−1^ EPS in submerged cultivation of *H. marmoreus*. Compared to our results, Chen et al. (2008) observed a lower EPS value (3.64 g∙L^−1^), but Alvandi et al. (2020) obtained a higher value of EPS (5.4 g∙L^−1^) through *F. fomentarius* fermentation under optimal culture conditions. Higher results for EPS yield of *G. applanatum* were reached in other studies: 2.2 g∙L^−1^ EPS by Lee et al. (2007) by applying different carbon/nitrogen ratios in the medium, and 9.1 g∙L^−1^ (Krupodorova 2011) in a whey-based medium.

Higher IPS values have been reported in several fungal species compared to our results: 50.39 ± 0.41 mg·g^−1^ in *P. igniarius* (Guo et al. 2010), 12.2–14.0% in *O. sinensis* (Poyedinok 2015), 7.5% in *G. lucidum* (Boromenskyi and Bisko 2020), and 4.3–6.5% in *H. erinaceus* (Mykchaylova et al. 2023). Lower IPS content was observed in *F. fomentarius* (2.86 g·L^−1^) (Chen et al. 2011), and *G. lucidum* (1.25 g·L^−1^) (Mahendran et al. 2012). Higher EPS values were reported for species such as *M. esculenta* (4.1–5.3 g∙L⁻^1^) (Taşkin et al. 2011), *P. eryngii* (7.27 g∙L⁻^1^) (Sun et al. 2014), *G. frondosa* (3.88 g∙L⁻^1^) (Osińska-Jaroszuk et al. 2015), *G. lucidum* (1.6–10.0 g∙L⁻^1^) (Krupodorova 2011; Kachrimanidou et al. 2023), and *C. militaris* (up to 5.713 g∙L⁻^1^) (Wang et al. 2019). Lower EPS values were recorded in species like *S. commune* (0.30–0.56 g∙L⁻^1^) (Ivanova et al. 2014) and *T. versicolor* (0.1–0.21 g∙L⁻^1^) (Osińska-Jaroszuk et al. 2015).

Polysaccharide production is influenced by grow conditions, fermentation type, and medium composition. For instance, EPS production in *G. lucidum* reached 8.28 g/100 mL in basal medium (Mahendran et al. 2012), while *F. betulina* strains produced 0.02–2.20 g∙L⁻^1^ EPS depending on the cultivation medium (Kizitska et al. 2024). Additives like Tween 80 and farnesol significantly enhanced IPS and EPS yields in *I. obliquus* (Xu et al. 2015), *P. igniarius* (Yang et al. 2021), and *T. versicolor* (Wang et al. 2017).

Fermentation systems also affect yields. *C. militaris* produced 2.27 g∙L⁻^1^ EPS in a flask and 5.713 g∙L⁻^1^ in a stirred tank reactor (Wang et al. 2019). Repeated-batch fermentation and co-culture approaches further enhanced EPS production, with co-cultured *G. lucidum* and *F. velutipes* producing EPS with lower cytotoxicity (Wu et al. 2021).

These findings emphasize the potential of the species studied for polysaccharide production. Optimizing cultivation conditions and employing advanced strategies can further enhance yields and expand their applications in nutraceuticals and medicine.

It should be noted that the species we studied exhibit significant potential for polysaccharide production, and understanding and implementing strategies to enhance their production could improve the productivity of these fungal species.

To the best of our knowledge, our screening studies contribute new insights into polysaccharide production in the poorly studied species *A. aurea*, *C. aegerita*, *H. myxotricha*, *L. luscina*, *L. schimeji*, *M. radiata*, *O. obducens*, and *P. litschaueri* under submerged cultivation, as this information has not been previously reported in the literature.

### Antioxidant activity

#### Free radical scavenging capacity

**Fig. 3:**
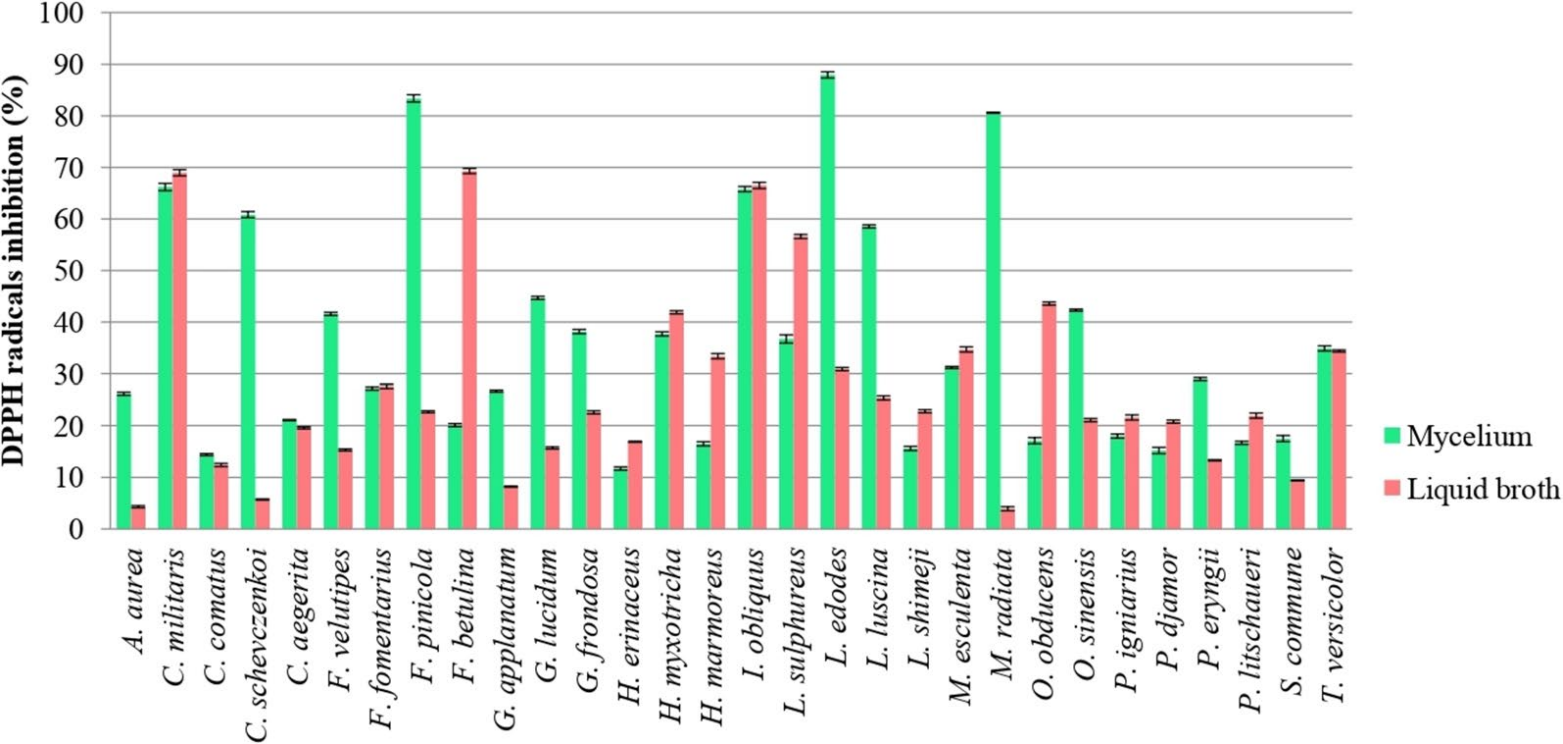
DPPH radical scavenging activity of ethyl acetate extracts from 30 fungal species. Significant differences were determined by one-way ANOVA using the Fisher’s LSD test. Data represent the mean ± standard deviation (SD), and differences were considered statistically significant at *p* < 0.05

The free-radical scavenging potential of EtOAc extracts from 30 macrofungal species was assessed. All species cultivated in GPYB medium, exhibited antiradical scavenging activity against DPPH radicals (Fig. 3). The DPPH inhibition values varied significantly from 4.30 ± 0.20 to 87.9 ± 0.62%, depending on the fungal species and analyzed sample type (mycelium or culture broth). In general, 50% of the fungal species demonstrated stronger antioxidant capacity in mycelium compared to the culture broth. The highest DPPH radical inhibition was observed in the mycelium of *L. edodes* (87.9 ± 0.62%), followed by *F. pinicola* (83.4 ± 0.75%) and *M. radiata* (80.6 ± 0.40%).

For culture broth samples, lower scavenging ability was observed for the same extract type and species (Fig. 3). However, the highest activity was noted in *F. betulina* (69.3 ± 0.55%), *C. militaris* (69.0 ± 0.62%), and *I. obliquus* (66.5 ± 0.63%). Notably, the mycelial antiradical activity of three xylotrophic species—*F. fomentarius* (27.3 ± 0.37%), *I. obliquus* (65.8 ± 0.57%), and *T. versicolor* (34.9 ± 0.50%), was comparable to that of their culture broth (27.6 ± 0.45%, 66.5 ± 0.63%, and 34.4 ± 0.26%, respectively). Species from the same genus, such as *G. applanatum*, *G. lucidum*, *F, betulina*, *F. pinicola*, *P. eryngii* and *P. djamor*, showed significant differences in DPPH inhibition between mycelium and culture broth. These findings contradict Dimitrijevic et al. (2015), who reported similar DPPH uptake capacities in fruiting bodies of *Boletus* spp. Conversely, Souilem et al. (2017) found comparable DPPH scavenging activities between mycelium and culture medium extracts of *P. eryngii*, reporting an EC_50_ value of 24.9 ± 0.3 mg∙mL^−1^, consistent with our results. Similarly, Reis et al. (2012) reported EC_50_ values of 25.40 ± 0.33 mg∙mL^−1^ for *P. eryngii* and 7.82 ± 0.56 mg∙mL^−1^ for *L. edodes* mycelium samples. Furthermore, Onar et al. (2016) tested *F. pinicola* EtOH extracts, finding lower DPPH inhibition (EC_50_ = 0.300 ± 0.005 mg∙mL^−1^ and 0.178 ± 0.004 mg∙mL^−1^, respectively) compared to our study. Uhrinová and Poľančíková (2018) reported significant effects of extraction procedures on the DPPH antioxidant activity of *O. sinensis* grown in different media, with ultrasound extraction achieving 84.8% activity at 10 mg·mL⁻^1^. Four years later, Flores et al. (2022) highlighted DPPH scavenging ability among *Pleurotus* spp., identifying *P. eryngii* var. *ferulae* as the most effective (EC_50_ = 0.886 ± 0.103 mg∙mL^−1^). Recent findings by Xiang et al. (2022) suggest that *F. velutipes* EPS contains proton-donating compounds stabilizing free radicals, with scavenging abilities of 59.55 ± 1.87%, 37.38 ± 2.02%, and 64.53 ± 3.02% for different fractions assigned as EPS, EPS-1 and EPS-2. MeOH extracts of *C. militaris* mycelium exhibited superior inhibition (85.37%) compared to our EtOAc extract (66.22 ± 0.73%), likely due to differences in extract polarity (Deshmukh and Lakshmi 2023). Dong et al. (2023) demonstrated 50% DPPH inhibition by polysaccharides from *P. igniarius* mycelia at 2 mg·mL⁻^1^. Bains et al. (2023) noted dose-dependent inhibition by *C. aegerita* MeOH extracts, ranging from 38.56 ± 0.11% (50 µg·mL⁻^1^) to 85.63 ± 0.12% (200 µg·mL⁻^1^), surpassing our findings.

Some studies reported lower DPPH values than ours for *T. versicolor* (IC_50_ = 0.52 mg·mL⁻^1^) (Jhan et al. 2016), *O. sinensis* (IC_50_ = 0.37–0.52 mg·mL⁻^1^) (Han et al. 2021; Chantnarin and Thirabunyanon 2023), and *P. igniarius* (IC_50_ = 0.78 mg·mL⁻^1^ and 6.84 ± 0.37 mg·mL⁻^1^) (Zhu and Li 2011; Dong et al. 2024).

These results provide a foundation for optimizing antioxidant activity. For example, studies on certain macromycetes, such as *Xylaria feejeensis* (Rebbapragada and Kalyanaraman 2016) and *F. pinicola* (Krupodorova et al. 2024) show that cultivation conditions can significantly enhance activity. The addition of Na₂SeO₃ (20 mg·L^−1^) to the culture medium significantly enhances antioxidant activity. For instance, adding 20 mg·L⁻^1^ Na₂SeO₃ increased *F. fomentarius* EPS antioxidant activity by 17.04% (Park and Lim 2024). A similar observation were observed in *P. djamor* (Velez et al. 2019).

### Principal Component Analysis (PCA) and correlation analysis

**Fig. 4:**
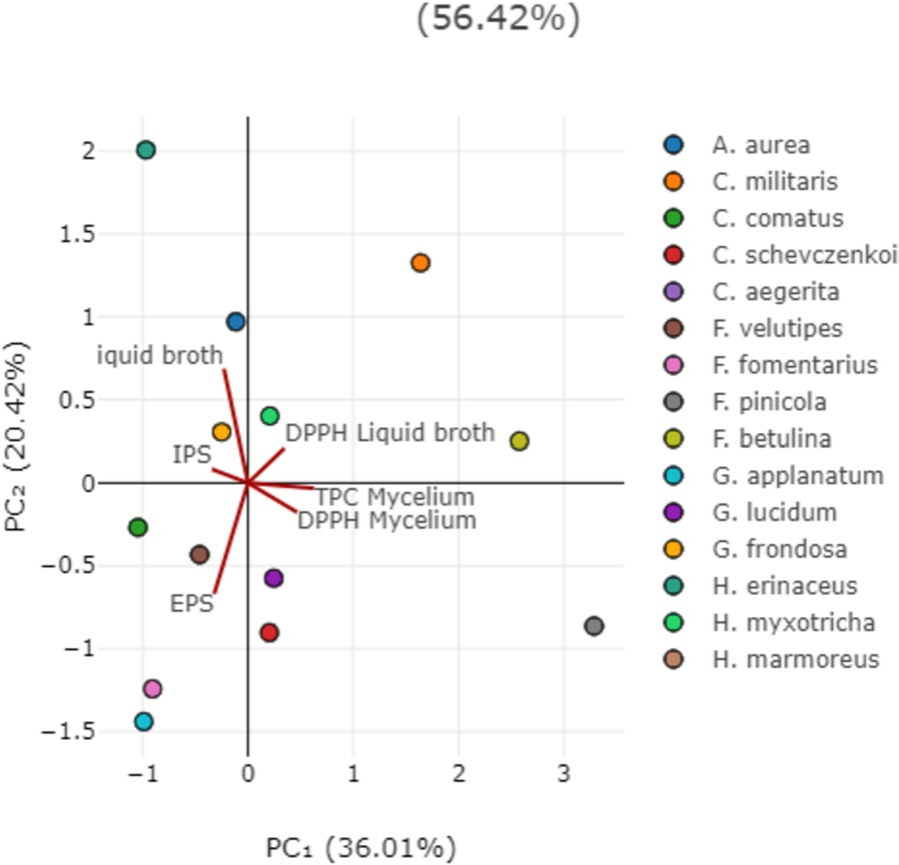
PCA analysis of all examined parameters in first 15 selected fungal species. The following abbreviations are used for the examined parameters: DPPH, radical scavenger capacity against 2,2-diphenyl-1-picrylhydrazyl radical, DPPH^⋅^; EPS, exopolysaccharides quantified in culture liquid broth; IPS, endopolysaccharides quantified in mycelium; TPC, total phenolic content.

**Fig. 5:**
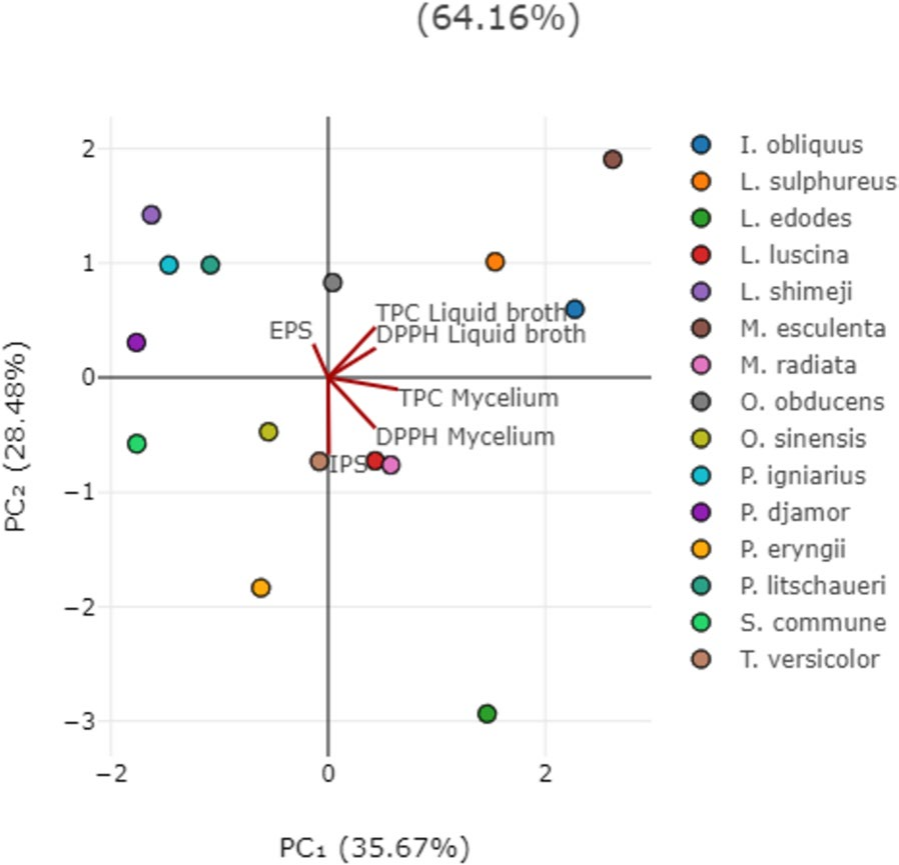
PCA analysis of all examined parameters in the second 15 selected fungal species. The following are the abbreviations of the examined parameters: DPPH, radical scavenger capacity against 2,2-diphenyl-1-picrylhydrazyl radical, DPPH^⋅^; EPS, exopolysaccharides quantified in culture liquid broth; IPS, endopolysaccharides quantified in mycelium; TPC, total phenolic content

PCA was employed to discern patterns and relationships among the quantified compounds (TPC, IPS, EPS) after submerged cultivation with the antioxidant activity of all investigated fungal species (Figs. 4 and 5).

In Fig. 4, the first two principal components, PC_1_ and PC_2_, accounted for 56.42% of the total variance, providing a substantial representation of the dataset. The PCA results emphasize the inherent variability within our dataset and provide insights into the primary factors driving the observed trends. A more distinct separation was observed in the horizontal plane of PC_2_, with TPC and IPS loading in the first quadrant and EPS in the third quadrant. TPC quantified in culture broth and IPS exhibited a high positive loading on both PCs, alongside the DPPH scavenging activity of liquid broth, suggesting their significant involvement in this activity. These variables separate *H. myxotricha*, *G. frondosa*, *A. aurea* and *C. militaris* from the other fungal species, suggesting that phenolics play a pivotal role in the observed antiradical activity. This observation is supported by the results presented in Figs. 1 and 2, as *C. militaris* stood out as one of the two most promising antioxidant agents, exhibiting DPPH inhibition of 69.0 ± 0.62% and TPC of 19.1 ± 0.65 mg GAE/g d.w. The correlation analysis results (Table S1) further validate this relationship.

Different pattern was noticed in Fig. 5, where the total variance was 64.16%, indicating that a significant portion of the dataset`s variability is captured by these components. The first axis (PC_1_) accounted for 35.7% and the second axis (PC_2_) for 28.5% of the total variability.

In contrast to Fig. 4, a stronger separation of the analyzed samples (35.67%) was observed in Fig. 5. In this figure, the DPPH scavenging activity and TPC results from liquid broth samples of 15 species are separated in the II quadrant, while the DPPH scavenging activity and TPC results from mycelium samples are separated in the IV quadrant, indicating specific patterns in the data. Species located in the II quadrant have higher values of DPPH and TPC compared to those in other quadrants. This is confirmed by the tested species, with *M. esculenta* standing out as the species with the highest TPC (Fig. 1). Other grouped species in the II quadrant are characterized by higher values of these particular measurements compared to mycelium samples. Mycelium samples separated in the IV quadrant suggest a connection between TPC and DPPH activity of *L. luscina*, *M. radiata*, and *S. commune*, along with IPS extracted from the mycelium.

These separations presented in Figs. 4 and 5 provide valuable insights into the relationships between TPC, EPS, IPS, and antiradical activity in the analyzed species, suggesting that the all 30 tested species in different quadrants have distinct profiles in terms of their antiradical activity, phenolic and polysaccharide contents. It can be generally concluded that there is a strong correlation between phenolics, which are the most promising antioxidant compounds from mushrooms (Rašeta et al. 2021, 2024a), and DPPH scavenging activity. Among the detected polysaccharides, EPS are not grouped with antiradical activity variables, with the exception of IPS, which did show a correlation. This lack of grouping indicates that EPS do not show a strong correlation with antiradical activity in the tested species, as confirmed by correlation analysis (Table S2).

The clustering observed in the score plot indicates the presence of potential subgroups with varying levels of tested activity and biologically active compounds. However, this grouping does not correlate with the species’ tropism or taxonomic classification. We propose that species-specific traits may influence the variations in detected compounds and biological activity, warranting further investigation into the biological factors contributing to these distinct patterns, as supported by Rašeta et al. (2024a).

*Fomitopsis pinicola* **and** *Lentinula edodes* **as the most potent antioxidant agents**.

**Fig. 6:**
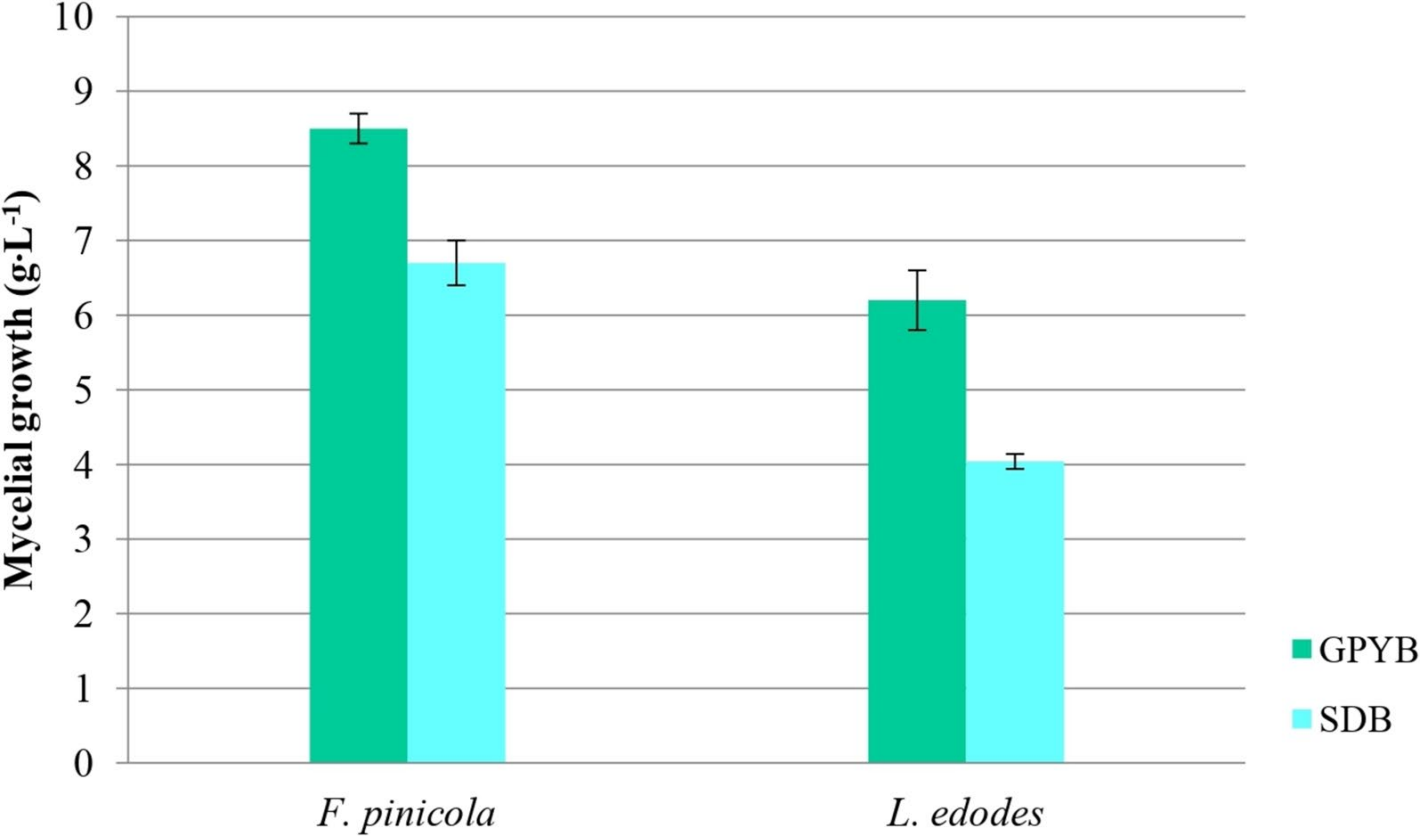
Fungal growth on different media. Significant differences were determined by one-way ANOVA using the Fisher’s LSD test. Data represent the mean ± standard deviation (SD), and differences were considered statistically significant at *p* < 0.05. The following abbreviations are used for the examined parameters: GPYB, glucose-peptone-yeast extract broth; SDB, Sabouraud dextrose broth

The empirical approach remains a crucial strategy and starting point for identifying promising fungal species. Based on the results of their antiradical activity and TPC content, we selected two xylotrophic Basidiomycetes, *F. pinicola* and *L. edodes*, for further investigation. The fungal growth of these species was assessed in terms of biomass production (Fig. 6), revealing differences in their biomass accumulation. Specifically, the GRYB medium supported better growth for both fungi compared to the SDB medium. *F. pinicola* produced more biomass (8.5 ± 0.2 and 6.7 ± 0.3 g∙L^−1^) than *L. edodes* in both media. For comparison, Choi et al. (2007) reported biomass yields of 7.9 g∙L^−1^ and 10.4 g∙L^−1^ for *F. pinicola* before and after optimizing cultivation conditions, respectively. Biomass yields for *L. edodes* under various cultivation conditions showed significant variability, ranging from 2.75 to 6.88 g·L⁻^1^ (Feng et al. 2010), 0.2 to 6.0 g·L⁻^1^ (Krupodorova et al. 2019), and 2.5 to 10.5 g·L⁻^1^ (Bisko et al. 2020).

Evaluating biomass is crucial for subsequent fermentation and production processes, as this metric indicates the potential and industrial viability of each species. In this context, *F. pinicola* demonstrates an advantage over *L. edodes* due to its greater capacity to generate biomass, regardless of the nutrient medium. We utilized two distinct media: semi-synthetic GPYB and the more organic, complex SDB. The enhanced growth of both fungi on the GPYB medium could be attributed to several factors, particularly the bioavailability and assimilation of carbon and nitrogen sources, their ratios, and the presence of mineral components. Glucose, a primary carbon source, is well known for its rapid breakdown and efficient energy supply to fungal organisms (Garraway and Evans 1984), aligning with the findings of numerous studies on basidiomycetes (Krupodorova et al. 2021). Addition, previous studies have also reported that certain mineral elements, such as magnesium and potassium, significantly stimulate mycelial growth (Jonathan and Fasidi 2001; Petre and Teodorescu 2009; Włodarczyk et al. 2020). The observed differences in biomass formation are likely influenced by the specific components of the nutrient medium and their effects on fungal metabolic pathways (Flores et al. 2022).

**Fig. 7:**
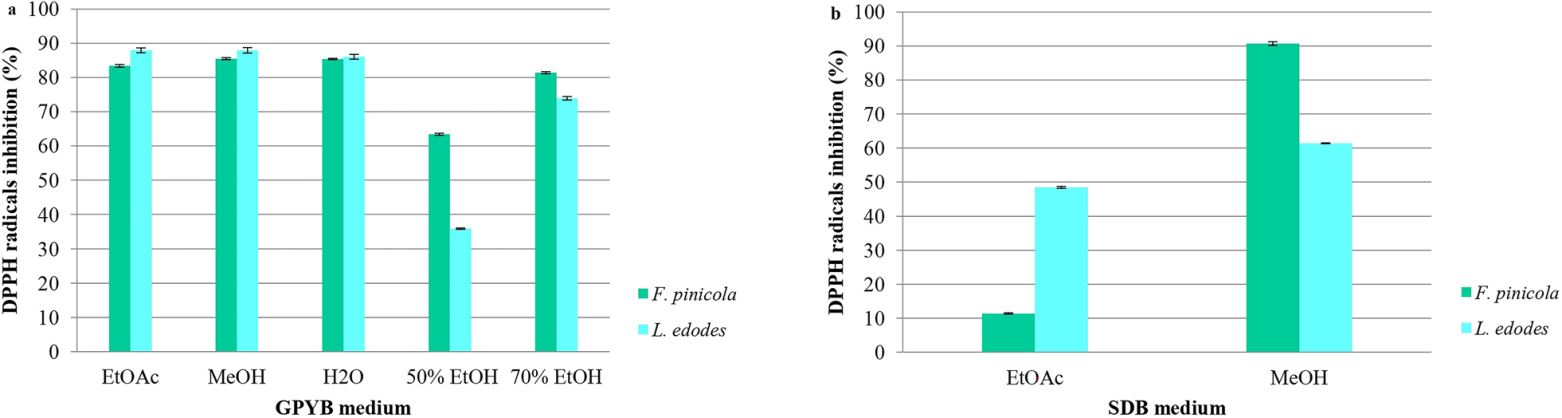
Effect of different solvents and media of fungi cultivation on antiradical scavenging activity; **a** GPYB medium and **b** SDB medium. Significant differences were determined by one-way ANOVA using the Fisher’s LSD test. Data represent the mean ± standard deviation (SD), and differences were considered statistically significant at *p* < 0.05. The following abbreviations are used for the examined parameters: EtOAc, ethyl acetate extract; MeOH, methanol extract; H_2_O, water extract; 50% EtOH, ethanolic extract was prepared using a mixture of ethanol and water in 50:50 ratio; 70% EtOH, ethanolic extract was prepared using a mixture of ethanol and water in 70:30 ratio; GPYB, glucose-peptone-yeast extract broth; SDB, Sabouraud dextrose broth

Using samples from *F. pinicola* and *L. edodes*, we evaluated the influence of culture medium and extractant on their antiradical activity (Fig. 7). All tested solvents successfully extracted antioxidant compounds capable of neutralizing the stable DPPH radical. The highest DPPH inhibition (90%) was observed in the MeOH extract of *F. pinicola* mycelium cultivated in the SDB medium. In comparison, Jiamworanunkul (2019) reported lower DPPH inhibition values for *L. edodes* mycelium, ranging from 31.42 ± 0.40% to 53.37 ± 0.44% in MeOH-EtOAc extracts. Notably, the differences in DPPH inhibition among EtOAc, MeOH, H_2_O, and 70% EtOH mycelial extracts were minimal for both macrofungi cultivated in the GPYB medium but more pronounced in those grown in the SDB medium.

Recently, our previous study (Krupodorova et al. 2024) also reported the presence of antiradical activity in the mycelium of *F. pinicola*. Additionally, antioxidant activity in the fruiting bodies of *F. pinicola* was observed in EtOH and MeOH extracts by Onar et al. (2016) and Kozarski et al. (2024), respectively. The antiradical activity of *L. edodes* mycelium has been analyzed in various studies using different solvents, including MeOH (Turło et al. 2010; Reis et al. 2012), H_2_O (Kalyoncu et al. 2010; Turło et al. 2010), EtOH (Kalyoncu et al. 2010; Suruga et al. 2022), chloroform (CHCl_3_) (Kalyoncu et al. 2010), and MeOH followed by EtOAc (Jiamworanunkul 2019). Kalyoncu et al. (2010) identified EtOH as a more effective extractant compared to CHCl_3_ and H_2_O, while Turło et al. (2010) observed superior results with MeOH over H_2_O.

In general, the GPYB medium promoted better antioxidant activity compared to SDB medium. The influence of the culture media on antioxidant activity has been observed in other studies (Barros et al. 2009; Dulay et al. 2016; Rebbapragada and Kalyanaraman 2016; Souilem et al. 2017; Darsih et al. 2019; Jiamworanunkul 2019; Flores et al. 2022; Krupodorova et al. 2022; Deshmukh and Lakshmi 2023). However, in our previous report, the SDB medium was more suitable for the antioxidant properties of EtOH extracts of *H. myxotricha* mycelium, assesed using Rel Assay TAS kits. The type of carbon source in the culture medium also influenced antioxidant activity (Barros et al. 2009). For example, *Leucopaxillus giganteus* mycelium showed higher antioxidant activity with glucose as the carbon source, while mannitol yielded lower activity. EtOAc extracts of *Volvariella volvacea* and *S.  commune* mycelium showed varying DPPH inhibition percentages depending on the liquid media used, with coconut water showing similar efficacy to potato dextrose broth (PDB) (Dulay et al. 2016). Similarly, MeOH extracts of *P.  eryngii* and *Suillus belinii* exhibited comparable efficacy against the DPPH radical on solid media (PDA and incomplete Melin-Norkrans medium), contrasting with the significant differences observed when using liquid media (Souilem et al. 2017). Potato dextrose yeast extract broth proved more effective than PDB, Czapek-Dox broth, and malt extract broth for DPPH inactivation of *Xylaria feejeensis* mycelium (Rebbapragada and Kalyanaraman 2016). Yeast extract sucrose broth (YES) supported higher DPPH inhibition in EtOAc mycelial extracts of *S. commune* and *Lentinus polychrous* compared to malt extract broth (MEB) and PDB. Conversely, MEB was more suitable for *G.  lucidum*, *L.  edodes*, and *L.  squarrosulus* (Jiamworanunkul 2019). MeOH extracts of *G.  lucidum* obtained after cultivation on PDB exhibited better antiradical effects than those cultivated in a natural medium from sugar corn (Darsih et al. 2019). The antiradical properties of 24 *Pleurotus* isolates were investigated using both DPPH and 2,2ʹ-azino-bis-3-ethylbenzothiazoline-6-sulfonic acid (ABTS) assays (Flores et al. 2022). For some samples, the culture medium did not affect the DPPH test, unlike the ABTS test, which was consistently influenced by the media used. The results obtained by Flores et al. (2022) underscore the species-specific nature of *Pleurotus* mycelia in acquiring antiradical activity depending on the nutrient medium and the method of antioxidant activity investigation.

**Fig. 8:**
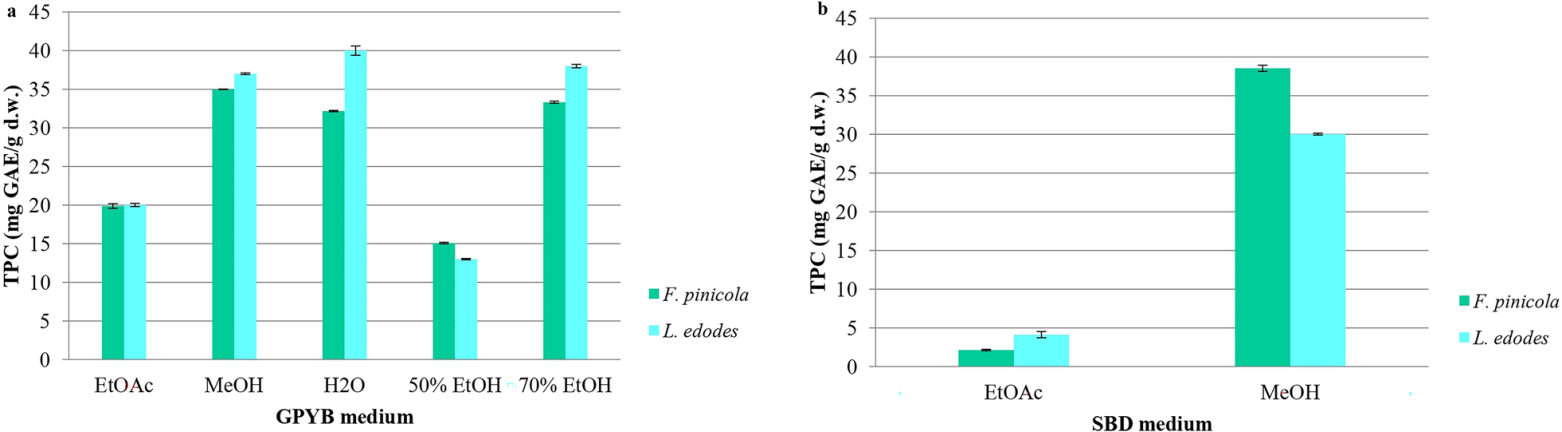
Effect of different solvents and media of fungi cultivation on total phenolic content. **a** GPYB medium and **b** SDB medium. Significant differences were determined by one-way ANOVA using the Fisher’s LSD test. Data represent the mean ± standard deviation (SD), and differences were considered statistically significant at *p* < 0.05. The following abbreviations are used for the examined parameters: TPC, total phenolic content; d.w., dry weight; GAE, gallic acid equivalents; EtOAc, ethyl acetate extract; MeOH, methanol extract; H_2_O, water extract; 50% EtOH, ethanolic extract was prepared using a mixture of ethanol and water in 50:50 ratio; 70% EtOH, ethanolic extract was prepared using a mixture of ethanol and water in 70:30 ratio; GPYB, glucose-peptone-yeast extract broth; SDB, Sabouraud dextrose broth

The influence of the culture medium and extractant on the TPC in the mycelium of *F. pinicola* and *L. edodes* was determined (Fig. 8). Higher TPC values of 40.0 ± 0.60 mg GAE/g d.w., 38.0 ± 0.10 mg GAE/g d.w., and 37.8 ± 0.10 mg GAE/g d.w. were found in the mycelia of *L. edodes* grown on GPYB and extracted with H_2_O, H_2_O-EtOH solution, and MeOH, respectively. The maximum TPC (38.5 ± 0.40 mg GAE/g d.w.) in *F. pinicola* mycelium was obtained in the MeOH extract when cultivated on SDB. To our knowledge, TPC in *F. pinicola* mycelium has not been reported in the literature. However, significantly higher TPC levels (133.1 ± 6.2 mg/g, 1278.0 ± 2.9 mg GAE/g, and 313.2 ± 5.8 mg GAE/g) have been recorded in *F. pinicola* fruiting bodies (Onar et al. 2016; Kozarski et al. 2024). Recent studies have identified vanillin and phenolic acids, including gallic, ellagic, protocatechuic, *p*-hydroxybenzoic, syringic, and chlorogenic acids, in *F. pinicola* fruiting bodies (Kozarski et al. 2024).

The determination of TPC in *L. edodes* mycelium aligns with previous reports. Reis et al. (2012) and Turło et al. (2010) reported lower TPC values (12.53 ± 0.30 mg GAE/g extract and 7.82–13.98 mg GAE/g) in MeOH and H_2_O extracts, respectively. Jiamworanunkul (2019) observed higher TPC (43.80 ± 0.38 mg GAE/g) in EtOAc extracts of *L. edodes* mycelium grown in MEB, but lower values (12.09 ± 0.69 and 19.00 ± 0.13 mg GAE/g) in PDB and YES media. Jimenez et al. (2023) reported even lower TPC (0.619–0.773 mg GAE/100 g d.w.) in *L. edodes* mycelium cultivated on eucalyptus biomass. Several phenolic acids have been identified in *L. edodes* mycelium, including protocatechuic acid and *p*-hydroxybenzoic acid (Reis et al. 2012), as well as homogentisic acid, gentisic acid, and caffeic acid (Vamanu et al. 2018).

The influence of the culture medium on TPC has been highlighted in prior studies. Rebbapragada and Kalyanaraman (2016) demonstrated that potato dextrose yeast extract broth was more effective than PDB, Czapek-Dox broth, and malt extract broth for achieving higher TPC in MeOH extracts of *Xylaria feejeensis* mycelium. Maximum TPC levels were observed in the mycelium of *G. lucidum*, *L. edodes*, and *L. squarrosulus* when cultivated in malt extract broth (MEB), whereas PDB and YES broths were more suitable for *L. polychrous* and *S. commune*, respectively (Jiamworanunkul 2019).

Pearson correlation analysis revealed a significant positive relationship between TPC and DPPH activity in fungal species (Table S3). According to Evans (1996), TPC showed strong correlation with DPPH activity in fungal mycelium (r^2^ = 0.6615) but weak correlation in fungal broth (r^2^ = 0.1192). Very strong correlations were found for *F. pinicola* (r^2^ = 0.8924) and strong correlations for *L. edodes* (r^2^ = 0.7143).

A positive correlation between TPC and DPPH was observed in *Xylaria polymorpha* and *X. longipes* mycelium and culture broth (Atamanchuk and Bisko 2023). However, no correlation between these two indicators was found in the mycelial extracts of *Agaricus bisporus*, *Armillaria mellea*, *A.  auricula-judae*, *G.  applanatum*, *G. lucidum*, *Laetiporus sulphureus*, *Lentinus tigrinus*, *Lycoperdon pyriforme*, *Phellinus linteus*, *P.  ostreatus*, *P. sajor-caju*, *Polyporus arcularius*, *Russula brevipes*, *S.  commune*, *Sparassis crispa*, *Spongipellis unicolor* (Prasad et al. 2015), and *G.  tuberculosum* (Campi et al. 2023).

## Conclusion

Recently, mushrooms have gained increasing recognition as natural producers of antioxidant agents, crucial for the prevention and treatment of diseases such as cancer, cardiovascular, diabetes, and neurodegenerative disorders. This study provides a comprehensive evaluation of the antioxidant potential and metabolite production in 30 macrofungal species, exploring possible correlations between total phenolic content (TPC), endo- and exopolysaccharides (IPS and EPS), and antioxidant activity.

The findings demonstrate significant variability in mycelial growth and metabolite production among the studied fungi, with the highest productivity exceeding 1000 mg·L⁻^1^·day⁻^1^ observed in *Ophiocordyceps sinensis*, *Pleurotus djamor*, and *Cordyceps militaris*. The screening studies conducted represent a crucial step in identifying promising producers of various metabolites. Notably, *Lentinula edodes* emerged as the most potent IPS producer, while *Hypsizygus marmoreus* exhibited the highest EPS yield, underscoring their potential applications in nutraceuticals and functional foods.

DPPH inhibition values varied widely, ranging from 4.30 to 87.9%, and TPC values ranged from 0.35 to 34.5 mg GAE/g d.w. Among the tested species, *L. edodes* and *Fomitopsis pinicola* exhibited the highest free radical scavenging potential (up to 90%) and the richest TPC (up to 40 mg GAE/g d.w.), highlighting them as prime candidates for further exploration as natural antioxidant sources. This study also emphasizes the critical role of cultivation media and extraction methods in determining the antioxidant activity and metabolite yield.

Significantly, antiradical scavenging activity and phenolic compound presence were reported for the first time in *Auriporia aurea*, *Hohenbuehelia myxotricha*, *Pseudospongipellis litschaueri*, and *Oxyporus obducens*. Additionally, TPC in the mycelium of *Lyopyllum shimeji*, *Lepista luscina*, and *Mensularia radiata* was documented for the first time, expanding the current understanding of these species.

The results establish a valuable database for future research and species selection in the development of mushroom-derived health products. The demonstrated relationships between phenolic content, polysaccharide production, and antioxidant activity suggest possible synergistic effects, enhancing the therapeutic potential of these natural products. These findings pave the way for further investigations and applications in nutraceutical development, highlighting the promising role of macrofungi as sources of bioactive compounds.

## Supplementary Information

Supplementary Material 1.

## Data Availability

All data generated or analyzed during this study are included in this published article.

## Abbreviations

ABTS: 2,2ʹ-Azino-bis-3-ethylbenzothiazoline-6-sulfonic acid
dH_2_O: Distilled water
CAGR: Compound Annual Growth Rate
DPPH: 1,1-Diphenyl-2-picryl-hydrazyl
EPS: Extracellular polysaccharides (exopolysaccharides)
EtOH: Ethanol
EtOAc: Ethyl acetate
GA: Gallic acid
GAE: Gallic acid equivalent
GPYA: Glucose peptone yeast agar
GPYB: Glucose peptone yeast broth
H_2_O: Water
MEB: Malt extract broth
MeOH: Methanol
IPS: Intracellular polysaccharides (endopolysaccharides)
PCA: Principal Component Analysis
PDB: Potato dextrose broth
P_IPS_: Productivity of endopolysaccharides
P_EPS_: Productivity of exopolysaccharides
P_TPC_: Productivity of total phenolic content
PCS: Plastic composite support
ROS: Reactive oxygen species
RNOS: Reactive nitrogen species
SD: Standard deviation
SDB: Sabouraud dextrose broth
TPC: Total phenolic content
YES: Yeast extract sucrose broth
