# Supplementary material for: Comparative evaluation of free radical scavenging activity and total metabolite profiles among 30 macrofungi species
Source: Bioresour Bioprocess. 2025 Feb 21;12(1):13. doi: 10.1186/s40643-025-00841-4 (PMC11845661; doi:10.1186/s40643-025-00841-4)
Supplement: Supplementary file 1 — Supplementary Material 1. [file 40643_2025_841_MOESM1_ESM.doc]

**Table S1.** Pearson’s correlation coefficient (r^2^) between productivity of mycelium and TPC, IPS and EPS of all tested species.

| Sample | Correlation coefficient (r^2^) | | |
| --- | --- | --- | --- |
|  | Mycelium/TPC | Mycelium/IPS | Mycelium/EPS |
| All tested species | -0.2424 | 0.6505 | 0.1339 |

Abbreviations: TPC – total phenolic content; IPS – total endopolysaccharides content; EPS – total exopolysaccharides content.

**Table S2.** Pearson’s correlation coefficient (r^2^) between DPPH radical scavenging activity and TPC, IPS and EPS of EtOAc extracts of all tested species.

| Sample | Correlation coefficient (r^2^) | | |
| --- | --- | --- | --- |
|  | DPPH/TPC | DPPH/IPS | DPPH/EPS |
| All tested species | 0.1192 | 0.1415 | -0.2129 |

Abbreviations: DPPH – 1,1-diphenyl-2-picryl-hydrazyl; TPC – total phenolic content; IPS – total endopolysaccharides content; EPS – total exopolysaccharides content.

**Table S3.** Pearson’s correlation coefficient (r^2^) between DPPH radical scavenging activity and TPC of *F. pinicola* and *L. edodes* tested mycelium extracts.

|  | DPPH | | | |
| --- | --- | --- | --- | --- |
|  | Fungal mycelium | Culture broth | *L. edodes* mycelium | *F. pinicola* mycelium |
| TPC | 0.6615 | 0.1192 | 0.7143 | 0.8924 |

Abbreviations: DPPH – 1,1-diphenyl-2-picryl-hydrazyl; TPC – total phenolic content.
